# Functionally diverse heteromeric traps for ligands of the transforming growth factor-β superfamily

**DOI:** 10.1038/s41598-021-97203-9

**Published:** 2021-09-15

**Authors:** Ravindra Kumar, Asya V. Grinberg, Huiming Li, Tzu-Hsing Kuo, Dianne Sako, Lavanya Krishnan, Katia Liharska, Jia Li, Rosa Grenha, Michelle C. Maguire, Steven D. Briscoe, R. Scott Pearsall, Brantley R. Herrin, Rajasekhar N. V. S. Suragani, Roselyne Castonguay

**Affiliations:** 1 Acceleron Pharma, Cambridge, MA USA; 2 Present Address: Dragonfly Therapeutics, Waltham, MA USA; 3 Present Address: Cellarity, Cambridge, MA USA

**Keywords:** Biotechnology, Drug discovery

## Abstract

Ligands of the transforming growth factor-β (TGF-β) superfamily are important targets for therapeutic intervention but present challenges because they signal combinatorially and exhibit overlapping activities in vivo. To obtain agents capable of sequestering multiple TGF-β superfamily ligands with novel selectivity, we generated soluble, heterodimeric ligand traps by pairing the extracellular domain (ECD) of the native activin receptor type IIB (ActRIIB) alternately with the ECDs of native type I receptors activin receptor-like kinase 4 (ALK4), ALK7, or ALK3. Systematic analysis of these heterodimeric constructs by surface plasmon resonance, and comparison with their homodimeric counterparts, revealed that each type I receptor partner confers a distinct ligand-binding profile to the heterodimeric construct. Additional characterization in cell-based reporter gene assays confirmed that the heterodimeric constructs possessed different profiles of signaling inhibition in vitro, which translated into altered patterns of pharmacological activity when constructs were administered systemically to wild-type mice. Our results detail a versatile platform for the modular recombination of naturally occurring receptor domains, giving rise to inhibitory ligand traps that could aid in defining the physiological roles of TGF-β ligand sets or be directed therapeutically to human diseases arising from dysregulated TGF-β superfamily signaling.

## Introduction

The transforming growth factor-β (TGF-β) superfamily is an attractive target for therapeutic intervention due to its wide-ranging roles in blood and tissue homeostasis^1–3^. However, the promiscuous interactions of superfamily ligands with their receptors and the frequently overlapping activities of ligands within subfamilies complicate efforts to manipulate signaling toward a desired outcome^4–6^. This challenge is exemplified by mammalian skeletal muscle biology, in which multiple activin-class ligands including activin A, activin B, and growth differentiation factor 8 (GDF8, or myostatin) are thought to act in a concerted manner with heteromeric combinations of four different superfamily receptors to regulate muscle fiber size^7,8^. To direct TGF-β superfamily signaling for therapeutic tissue regeneration and repair, it is therefore necessary to develop tools for the concomitant control of defined ligand sets within the wider space of related superfamily signaling molecules.

**Figure 1:**
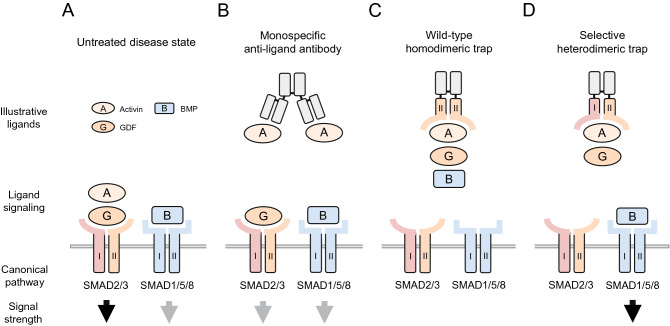
Hypothetical advantage of inhibiting functional subgroups of TGF-β superfamily ligands using selective heterodimeric traps. Human disease states are often characterized by imbalance between mutually antagonistic SMAD2/3 and SMAD1/5/8 signaling pathways. (**A**) In a hypothetical untreated disease state, excessive SMAD2/3 signaling (black arrow) occurs in combination with diminished SMAD1/5/8 signaling (grey arrow). A successful therapeutic intervention would restore normal balance between these two branches, and in some cases boost SMAD1/5/8 signaling, through selective inhibition of multiple SMAD2/3-activating ligands. (**B**) A monospecific anti-ligand antibody sequesters one SMAD2/3 ligand, but this partial inhibition might be inadequate to fully restore normal SMAD pathway balance. (**C**) A homodimeric ligand trap based on receptor ECD-Fc fusion might bind multiple ligands of both SMAD signaling branches, causing deleterious loss of SMAD1/5/8 signal. (**D**) A selective heterodimeric trap, engineered to target multiple ligands of the SMAD2/3 pathway but not of the SMAD1/5/8 pathway, could normalize SMAD pathway balance through robust inhibition of SMAD2/3 signaling while preserving SMAD1/5/8 signaling and potentially promoting it indirectly through disinhibition (black arrow). I, type I receptor or its ECD; II, type II receptor or its ECD. Ligands and endogenous receptors are depicted as monomeric for clarity.

In humans and other mammals, more than 30 secreted superfamily ligands converge upon a more limited repertoire of targets including seven type I transmembrane receptors, known as activin receptor-like kinases (ALKs), and five type II transmembrane receptors. Although the molecular mechanisms differ across ligand subfamilies^9,10^, binding of a dimeric superfamily ligand with the receptor extracellular domain (ECD) triggers assembly of a heterotetrameric receptor signaling complex containing two type I and two type II receptors. In the canonical pathway, the activated receptor complex in turn regulates SMAD transcription factors, which transduce signals from the receptor intracellular kinase domains to the cell nucleus^11^. Importantly, signal can propagate through distinct SMAD2/3 or SMAD1/5/8 branches based on the identity of the type I receptor incorporated in the signaling complex (Fig. 1A). Because these two classes of transcription factors elicit different and often opposing transcriptional responses^12–14^, a therapeutic compound that targets one or the other selectively could offer advantages over less discriminate approaches.

One effective strategy to inhibit multiple TGF-β superfamily ligands involves the use of homodimeric fusion proteins, in which an immunoglobulin fragment crystallizable (Fc) domain is attached to the ECD of either a type I or type II receptor to generate a soluble ligand trap. In contrast to monoclonal antibodies, which would be expected to sequester only ligands that share a targeted epitope (Fig. 1B), such receptor-based constructs concurrently neutralize groups of ligands and block their combined actions (Fig. 1C). Because dimeric superfamily ligands engage typically in symmetric interactions with two copies each of type I and type II receptors, coordinate binding by paired ECDs within dimeric trap molecules is thought to enhance ligand sequestration in comparison with constructs based on a single chain. Notable examples include homodimeric traps based on the native activin receptor type IIA (ActRIIA, encoded by *ACVR2A*)^15–17^ and ActRIIB (encoded by *ACVR2B*)^18,19^. These type II receptors possess relatively broad ligand-binding specificities and can mediate either SMAD2/3 or SMAD1/5/8 signaling depending on type I receptor pairing and on binding competition between high-affinity activin-class ligands and lower-affinity ligands such as bone morphogenetic proteins (BMPs)^6,20,21^. As a clinical consequence, the homodimeric ActRIIB-Fc fusion protein increases skeletal muscle mass by sequestering the SMAD2/3-pathway ligands activin A, activin B, GDF8, and GDF11 but is also thought to cause undesirable vascular effects by sequestering the SMAD1/5/8-pathway ligand BMP9^22^.

The TGF-β superfamily-based homodimeric traps generated to date sequester ligand combinations from a limited domain of ligand space, one that could be further exploited for therapeutic opportunities by developing additional, structurally diverse receptor constructs with altered binding properties. Here we present a novel molecular platform to selectively generate heterodimeric receptor combinations, which are analogous to half of a native heterotetrameric TGF-β receptor signaling complex. As proof-of-principle for this platform, we paired the ECD of the well-characterized type II receptor ActRIIB^21,23^ alternately with ECDs from type I receptors of either the SMAD2/3 signaling branch (ALK4 and ALK7) or the SMAD1/5/8 signaling branch (ALK3) (Fig. 1D). Our characterization studies indicate that each type I receptor partner confers a distinct ligand-binding profile as well as a distinctive pattern of pharmacological activity in vitro and in vivo. This strategy for modular recombination of naturally occurring receptor elements can thus generate diverse ligand traps that possess novel and potentially useful biological activities with limited off-target effects.

## Results

### Generation of selective heterodimeric ligand traps

**Figure 2:**
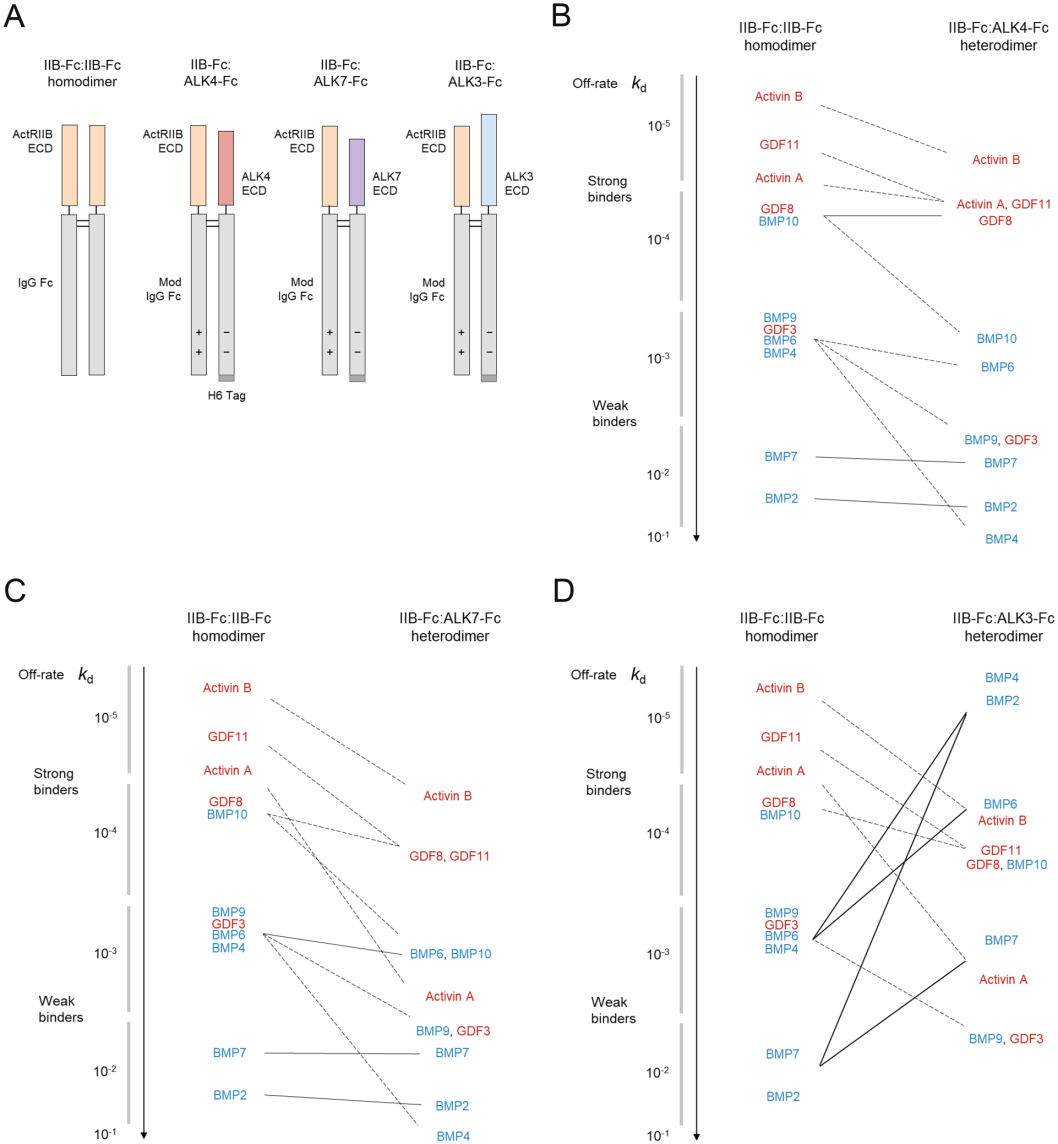
Profiles of ligand dissociation kinetics for heterodimeric ligand traps vary with type I receptor component. (**A**) Domain schematics depicting variants based on naturally occurring pairings of TGF-β superfamily type I receptor ECDs with ActRIIB ECD. The Fc domains of heterodimeric fusion proteins are modified (Mod) to promote desired pairing of monomers during cellular production. H6, histidine hexamer. (**B–D**) Ligand sequestration profiles for ActRIIB-Fc-based heterodimeric traps vary markedly with the identity of the partnered type I receptor ECD. Semi-schematic graphs depict the change in off-rate (*k*_d_) of key ligands, as determined by surface plasmon resonance (see Table 1 for exact values), when one ECD in the IIB-Fc:IIB-Fc homodimer is replaced with ALK4 (**B**), ALK7 (**C**), or ALK3 (**D**).

We generated ligand-trapping fusion proteins in Chinese hamster ovary (CHO) cells by expression of ECD-Fc polypeptide chains, which become linked covalently by disulfide bonds between the Fc domains to form stable dimers with antibody-like pharmacokinetic properties^24^. Whereas homodimeric fusion proteins of this type comprise a pair of identical polypeptide chains (Fig. 2A, IIB-Fc:IIB-Fc), heterodimeric constructs require preferential pairing of different ECD chains. We therefore engineered differentially charged amino acid substitutions into the Fc domains of intended polypeptide pairs to produce complementary electrostatic interactions that favor heterodimeric chain pairing over homodimeric pairing^25–27^. In addition, a hexameric histidine tag was included at the carboxy terminus of the type I receptor chain in each heterodimeric construct to facilitate its purification (Fig. 2A, H6 Tag). In this manner, we produced a set of three heterodimeric fusion proteins (IIB-Fc:ALK3-Fc, IIB-Fc:ALK4-Fc, and IIB-Fc:ALK7-Fc) in which a human ActRIIB ECD-Fc fusion was alternately paired with polypeptide chains incorporating the ECDs from human ALK3 (*BMPR1A*), human ALK4 (*ACVR1B*), or human ALK7 (*ACVR1C*), respectively (Fig. 2A). For comparison, we also produced the analogous homodimers IIB-Fc:IIB-Fc, ALK3-Fc:ALK3-Fc, ALK4-Fc:ALK4-Fc, and ALK7-Fc:ALK7-Fc.

### Ligand binding profiles of selective heterodimeric ligand traps in vitro

We used surface plasmon resonance (SPR) analysis to systematically measure the ligand binding kinetics of these homodimeric and heterodimeric fusion proteins based on a panel of 11 homodimeric ligands from the TGF-β superfamily (Table 1, Supplemental Fig. S1, Supplemental Table S1). Ligands selected for analysis included five that activate SMAD2/3 signaling (activin A, activin B, GDF3, GDF8, and GDF11) and six that activate SMAD1/5/8 signaling (BMP2, BMP4, BMP6, BMP7, BMP9, and BMP10)^4,28^. The eponymous superfamily ligands TGF-β1, TGF-β2, and TGF-β3 were notably excluded from analysis as they are thought to bind predominantly, if not exclusively, to TGFBRII and ALK5 but not to the receptors tested here^6,10,29^.

**Table 1:** Kinetic parameters of ligand binding by Fc-fusion protein constructs as determined by SPR.

Ligand	Kinetic parameter	Reference	Heterodimers			Homodimers		
		IIB-Fc:IIB-Fc	IIB-Fc:ALK4-Fc	IIB-Fc:ALK7-Fc	IIB-Fc:ALK3-Fc	ALK4-Fc:ALK4-Fc	ALK7-Fc:ALK7-Fc	ALK3-Fc:ALK3-Fc
**Activin A**	*k*_a_ (× 10^6^ M^−1^ s^−1^)	7.8 ± 0.1	7.4 ± 1.0	**20.0 ± 0.8**	**44.7 ± 0.9**	0.2 ± 0.0	NB	NB
	*k*_d_ (× 10^–6^ s^−1^)	84.5 ± 4.2	139 ± 34.3	**8150 ± 266**	**7330 ± 327**	> 10,000		
	*K*_D_ (pM)	10.9 ± 0.5	18.4 ± 2.5	**410 ± 28.5**	**164 ± 4.8**	> 10,000		
**Activin B**	*k*_a_ (× 10^6^ M^−1^ s^−1^)	5.7 ± 0.2	5.3 ± 0.3	7.4 ± 0.1	4.8 ± 0.2	NB	NB	NB
	*k*_d_ (× 10^–6^ s^−1^)	21.9 ± 2.6	80.7 ± 10.3	114 ± 12.3	335 ± 7.8			
	*K*_D_ (pM)	3.8 ± 0.4	15.3 ± 1.9	15.3 ± 1.4	69.6 ± 2.0			
**GDF3**	*k*_a_ (× 10^6^ M^−1^ s^−1^)	2.9 ± 0.1	**1.1 ± 0.0**	**1.1 ± 0.0**	**0.9 ± 0.0**	NB	NB	NB
	*k*_d_ (× 10^–6^ s^−1^)	1880 ± 34.6	** > 10,000**	** > 10,000**	** > 10,000**			
	*K*_D_ (pM)	640 ± 6.7	** > 10,000**	** > 10,000**	** > 10,000**			
**GDF8**	*k*_a_ (× 10^6^ M^−1^ s^−1^)	3.2 ± 0.1	2.8 ± 0.1	3.0 ± 0.1	2.7 ± 0.1	NB	NB	NB
	*k*_d_ (× 10^–6^ s^−1^)	125 ± 3.3	236 ± 1.3	637 ± 2.9	696 ± 6.8			
	*K*_D_ (pM)	39.1 ± 0.7	83.7 ± 2.0	212 ± 2.7	256 ± 6.9			
**GDF11**	*k*_a_ (× 10^6^ M^−1^ s^−1^)	19.4 ± 0.3	15.2 ± 0.3	13.5 ± 0.2	13.2 ± 0.3	0.5 ± 0.2	NB	NB
	*k*_d_ (× 10^–6^ s^−1^)	75.6 ± 2.2	164 ± 3.2	642 ± 10.7	641 ± 2.4	> 10,000		
	*K*_D_ (pM)	3.9 ± 0.2	10.8 ± 0.4	47.4 ± 0.0	48.5 ± 1.3	> 10,000		
**BMP2**	*k*_a_ (× 10^6^ M^−1^ s^−1^)	**1.0 ± 0.0**	**0.4 ± 0.0**	**0.4 ± 0.0**	12.3 ± 0.5	NB	NB	1.5 ± 0.0
	*k*_d_ (× 10^–6^ s^−1^)	** > 10,000**	** > 10,000**	** > 10,000**	35.5 ± 1.8			260 ± 12.1
	*K*_D_ (pM)	** > 10,000**	** > 100,000**	** > 100,000**	2.9 ± 0.3			177 ± 10.1
**BMP4**	*k*_a_ (× 10^6^ M^−1^ s^−1^)	0.6 ± 0.0	NB	NB	3.0 ± 0.0	NB	NB	0.4 ± 0.0
	*k*_d_ (× 10^–6^ s^−1^)	3520 ± 193			12.6 ± 0.7			88.3 ± 4.9
	*K*_D_ (pM)	5430 ± 95.8			4.1 ± 0.2			202 ± 11.9
**BMP6**	*k*_a_ (× 10^6^ M^−1^ s^−1^)	7.8 ± 0.1	7.6 ± 0.2	5.9 ± 0.5	4.6 ± 0.2	NB	NB	0.6 ± 0.0
	*k*_d_ (× 10^–6^ s^−1^)	2730 ± 55.7	5730 ± 249	4240 ± 307	214 ± 11.0			5690 ± 128
	*K*_D_ (pM)	348 ± 4.2	758 ± 15.2	715 ± 8.4	46.0 ± 1.6			9030 ± 58.3
**BMP7**	*k*_a_ (× 10^6^ M^−1^ s^−1^)	**0.8 ± 0.0**	**0.8 ± 0.0**	**0.7 ± 0.0**	11.9 ± 0.2	NB	NB	NB
	*k*_d_ (× 10^–6^ s^−1^)	** > 10,000**	** > 10,000**	** > 10,000**	1970 ± 50.2			
	*K*_D_ (pM)	** > 10,000**	** > 10,000**	** > 10,000**	165 ± 2.4			
**BMP9**	*k*_a_ (× 10^6^ M^−1^ s^−1^)	12.4 ± 0.7	**8.9 ± 0.6**	**7.1 ± 0.9**	**9.7 ± 0.3**	NB	NB	NB
	*k*_d_ (× 10^–6^ s^−1^)	1100 ± 6.6	** > 10,000**	** > 10,000**	** > 10,000**			
	*K*_D_ (pM)	93.3 ± 4.3	**1360 ± 77.9**	**2300 ± 145**	**1760 ± 30.5**			
**BMP10**	*k*_a_ (× 10^6^ M^−1^ s^−1^)	18.6 ± 0.8	41.8 ± 0.3	44.5 ± 1.0	21.5 ± 0.1	NB	NB	NB
	*k*_d_ (× 10^–6^ s^−1^)	121 ± 2.6	2800 ± 35.3	3140 ± 60.6	719 ± 11.0			
	*K*_D_ (pM)	6.5 ± 0.4	67.0 ± 1.1	70.6 ± 1.0	33.5 ± 0.7			

Values are means ± SEMs (n = 3 independent experiments). NB, no binding. Bold cells indicate values obtained by bivalent analyte modeling, all others by 1:1 modeling (see “Methods”). Activin-class ligands and GDF3 are SMAD2/3-pathway activators and BMPs are SMAD1/5/8-pathway activators.

Characterization of ligand-binding kinetics revealed that the three heterodimeric constructs differed markedly from the IIB-Fc:IIB-Fc homodimer and among themselves. Kinetic parameters for these constructs as well as homodimeric counterparts based on ALK3, ALK4, and ALK7 are shown in Table 1, and Fig. 2 depicts pronounced differences among the heterodimeric constructs regarding the ligand dissociation rate constant (*k*_d_), or off-rate, an important parameter describing duration of ligand sequestration. A useful way to interpret the diagrams in Fig. 2B–D is by the overall pattern of lines depicting change in off-rate for the ligands, and for a given comparison between IIB-Fc:IIB-Fc and heterodimer the number of line intersections provides an indication of the degree to which the off-rate profile is altered by ECD replacement.

Based on off-rates obtained by SPR analysis, the heterodimeric construct containing an ALK4 ECD (IIB-Fc:ALK4-Fc) retained relatively strong binding to most SMAD2/3-pathway ligands but showed markedly reduced preference for BMP4, BMP9, BMP10, and GDF3 compared with the parent ActRIIB-Fc homodimer (Fig. 2B). This heterodimeric construct was a dramatically more effective trap than the corresponding ALK4-Fc:ALK4-Fc homodimer (Table 1), consistent with the relatively weak binding by activin-class ligands to their cognate type I receptors without an accompanying type II receptor^30,31^.

The heterodimer containing an ALK7 ECD (IIB-Fc:ALK7-Fc) displayed an off-rate profile resembling features of the IIB-Fc:ALK4-Fc profile. Like IIB-Fc:ALK4-Fc, IIB-Fc:ALK7-Fc retained the overall preference for SMAD2/3-pathway ligands of its parent homodimeric construct, IIB-Fc:IIB-Fc. With the exception of activin A, the rank order of ligand off-rates remained largely the same for IIB-Fc:ALK7-Fc as for IIB-Fc:IIB-Fc, but most off-rate values were increased approximately tenfold, thereby lessening the sequestration effectiveness for the weaker-binding ligands in particular. These included BMP4, BMP9, BMP10, and GDF3, but especially activin A, which displayed a disproportionately large increase in off-rate (approximately 100-fold) compared with IIB-Fc:IIB-Fc (Fig. 2C). As expected, the IIB-Fc:ALK7-Fc heterodimeric construct was a much more effective trap than the corresponding type I homodimeric construct, ALK7-Fc:ALK7-Fc (Table 1).

In marked contrast, the heterodimeric construct containing ALK3—a key regulator of SMAD1/5/8 signaling—bound with greatly increased strength to BMP2, BMP4, BMP6, and BMP7 but with lower strength to the cohort of SMAD2/3-pathway ligands (Fig. 2D). For all three heterodimeric ligand traps, BMP9 and BMP10 exhibited faster off-rates (Fig. 2B–D), consistent with the observation that these ligands signal primarily through receptor complexes containing ALK1 as the type I receptor^32^ and not through ALK4, ALK7, or ALK3. Together, these SPR data demonstrate that each combination of receptor ECDs exhibits a distinct binding profile in vitro, and by replacing one arm of the ActRIIB homodimer with a type I receptor ECD it is possible to modify binding properties to preferentially sequester different subsets of TGF-β superfamily ligands.

### Inhibitory potency of selective heterodimeric ligand traps in vitro

We next investigated whether the altered ligand binding profiles observed for the heterodimeric ligand traps in SPR assays translate to novel patterns of signal inhibition in a cell-based reporter gene assay. The three heterodimeric and four homodimeric trap constructs were evaluated for their ability to inhibit signal transduction initiated by four SMAD2/3-pathway ligands in the activin class (activin A, activin B, GDF8, and GDF11) and two SMAD1/5/8-pathway ligands (BMP9 and BMP10). The SMAD2/3-pathway ligands were tested using A204 cells transfected with a SMAD2/3-responsive luciferase reporter plasmid (utilizing a CAGA12 promoter^33^), whereas the SMAD1/5/8 ligands were tested using T98G cells transfected with a SMAD1/5/8-responsive reporter (utilizing a BRE promoter^34^).

**Figure 3:**
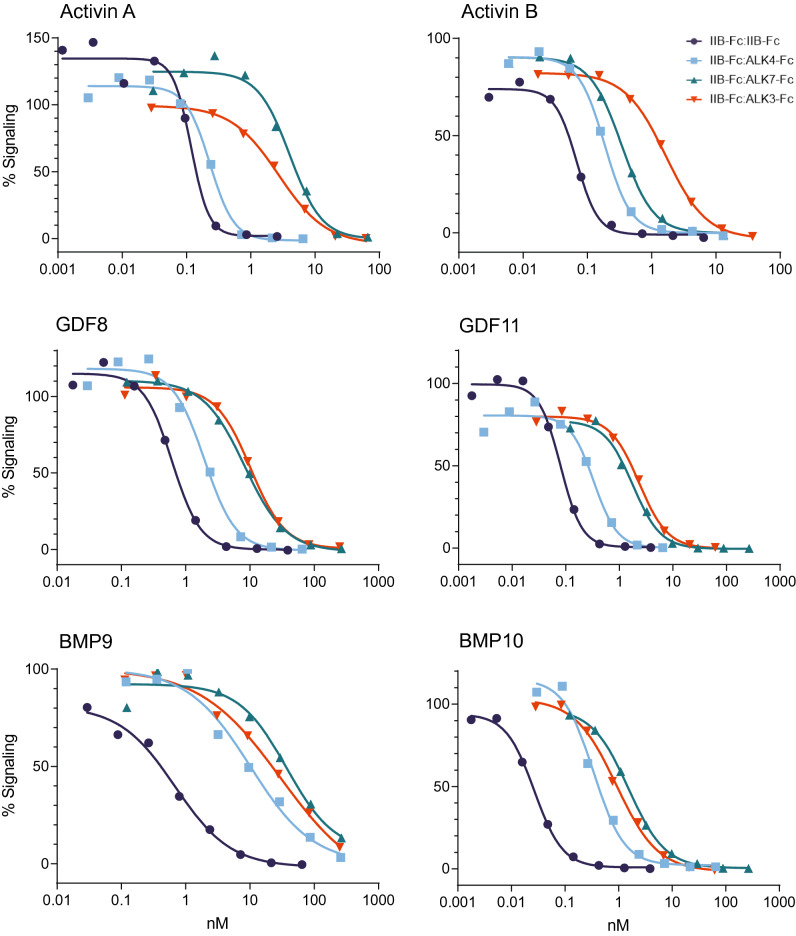
The type I receptor component in heterodimeric traps impacts signaling activity in a cellular context in vitro. Representative curves from assays conducted with a pSMAD2/3-responsive luciferase reporter gene in A204 cells (activin A, activin B, GDF8, and GDF11) or a pSMAD1/5/8-responsive luciferase reporter gene in T98G cells (BMP9 and BMP10) are depicted. Data from three independent experiments were used to determine mean values for half-maximal inhibitory concentration (IC_50_) shown in Table 2. Signaling level of 100% was defined as reporter activity for cells treated with ligand alone and 0% as reporter activity for cells treated with media alone. Values above or below 100% at the lowest ligand trap concentrations therefore reflect experimental variability observed for this representative replicate.

Table 2 lists mean values for half-maximal inhibitory concentration (IC_50_) determined in these assays and Fig. 3 depicts representative inhibition curves for each ligand-construct combination for which an IC_50_ value could be determined. The three heterodimeric constructs inhibited signaling by BMP9 and BMP10 with similarly reduced potency compared with the IIB-Fc:IIB-Fc homodimer, with reductions of approximately 15-to-54-fold for BMP9 and 23-to-67-fold for BMP10, but exhibited diverse signal inhibition patterns for the activin-class ligands. For activin A inhibition, IIB-Fc:ALK4-Fc (IC_50_ = 0.19 ± 0.04 nM) displayed potency comparable to that of IIB-Fc:IIB-Fc (IC_50_ = 0.11 ± 0.01 nM), whereas IIB-Fc:ALK7-Fc (IC_50_ = 2.86 ± 0.60 nM) and IIB-Fc:ALK3-Fc (IC_50_ = 2.62 ± 0.15 nM) showed reduced inhibitory potencies consistent with their faster ligand off-rate profiles determined by SPR (Fig. 2C). For activin B inhibition, IIB-Fc:ALK3-Fc (IC_50_ = 1.65 ± 0.02 nM) exhibited the greatest reduction in potency compared with IIB-Fc:IIB-Fc (IC_50_ = 0.06 ± 0.01 nM), whereas IIB-Fc:ALK4-Fc (IC_50_ = 0.16 ± 0.01 nM) and IIB-Fc:ALK7-Fc (IC_50_ = 0.26 ± 0.05 nM) constructs were intermediate in potency. The inhibition profiles of GDF8 and GDF11 resembled each other. In each case, IIB-Fc:IIB-Fc was the most potent inhibitor, IIB-Fc:ALK7-Fc and IIB-Fc:ALK3-Fc were least potent, and IIB-Fc:ALK4-Fc was intermediate in potency. As expected, the homodimeric type I constructs were uniformly poor inhibitors of signaling by these six ligands in the reporter gene assay (Table 2).

**Table 2:** Mean IC_50_ values for ligand inhibition by Fc-fusion constructs in a reporter-gene assay.

Ligand	Reference	Heterodimers			Homodimers		
	IIB-Fc:IIB-Fc	IIB-Fc:ALK4-Fc	IIB-Fc:ALK7-Fc	IIB-Fc:ALK3-Fc	ALK4-Fc:ALK4-Fc	ALK7-Fc:ALK7-Fc	ALK3-Fc:ALK3-Fc
**Activin A**	0.11 ± 0.01	0.19 ± 0.04	2.86 ± 0.60	2.62 ± 0.15	> 100	ND	ND
**Activin B**	0.06 ± 0.01	0.16 ± 0.01	0.26 ± 0.05	1.65 ± 0.02	ND	ND	ND
**GDF8**	0.76 ± 0.10	2.04 ± 0.04	9.15 ± 1.17	10.78 ± 0.31	ND	ND	ND
**GDF11**	0.09 ± 0.01	0.34 ± 0.02	2.34 ± 0.67	2.42 ± 0.07	ND	ND	ND
**BMP9**	0.57 ± 0.06	8.47 ± 1.09	31.02 ± 4.25	23.40 ± 3.34	ND	ND	ND
**BMP10**	0.03 ± 0.00	0.70 ± 0.19	2.01 ± 0.29	1.10 ± 0.14	ND	ND	ND

Values are means ± SEMs (n = 3), expressed in nM. ND, no inhibition determined at the highest experimental concentration for each molecule tested.

Together, these cell-based data indicate that each heterodimeric combination of receptor ECDs exhibits a novel pattern of signal inhibition when compared among themselves and with their corresponding homodimeric constructs. Furthermore, the directionality of IC_50_ shifts observed by this complementary assay was consistent with the heterodimer-specific ligand binding profiles obtained by SPR, particularly the increased off-rates observed for the tested ligands.

### Pharmacological activity profiles of selective heterodimeric ligand traps in vivo

We characterized the activity of systemically administered heterodimers (IIB-Fc:ALK4-Fc, IIB-Fc:ALK7-Fc, and IIB-Fc:ALK3-Fc) in wild-type C57BL/6 mice in comparison with the IIB-Fc:IIB-Fc homodimer. For mice receiving each individual ligand trap, we measured total body weight, skeletal muscle weight, body fat as a percentage of body weight, and bone mineral density—parameters known to be regulated by TGF-β superfamily signaling^1–3^. Drug or vehicle control (phosphate buffered saline, PBS) were administered by subcutaneous (s.c.) injection twice weekly for 28 days. To assess the biologic effects of treatment on multiple tissue composition, body fat and bone mineral density were measured by nuclear magnetic resonance (NMR) imaging and dual-energy x-ray absorptiometry (DXA), respectively, at baseline (day -1) and day 27. The gastrocnemius muscle, which responds strongly to manipulation of TGF-β superfamily signaling^8,35^, was chosen as a representative muscle and weighed at day 28. Control experiments indicated that replacement of a human Fc domain with its murine counterpart does not affect activity of a IIB-Fc:IIB-Fc homodimeric construct tested in mice for 8 weeks (Supplemental Fig. S2).

**Figure 4:**
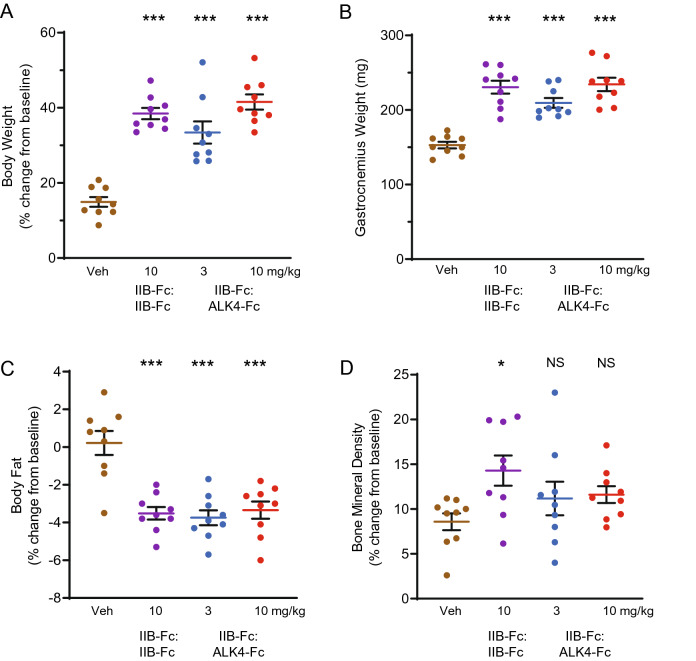
Activity of IIB-Fc:ALK4-Fc heterodimer in wild-type mice. Eight-week-old wild-type C57BL/6 mice (*n* = 9 per group) were injected s.c. with IIB-Fc:IIB-Fc (10 mg/kg), IIB-Fc:ALK4-Fc (either 3 mg/kg or 10 mg/kg), or vehicle control (PBS) twice weekly for 28 days. (**A**) Percentage change in mouse total body weight from day -1 to day 28. (**B**) Weight of the gastrocnemius muscle isolated by dissection on day 28. (**C**) Percentage change in total fat mass normalized to body weight from day -1 to day 27 as assessed by NMR. (**D**) Percentage change in bone mineral density from day -1 to day 27 as assessed by DXA. Data are means ± SEM. See Supplemental Table S2 for associated statistics. *Veh* vehicle; *mg/kg* milligrams per kilogram; *NS* not significant. *P < 0.05, ***P < 0.001 vs. vehicle.

In wild-type mice, the IIB-Fc:ALK4-Fc heterodimer displayed an activity profile similar to that of IIB-Fc:IIB-Fc, which is known to alter body composition^36^. Specifically, treatment with IIB-Fc:ALK4-Fc produced a dose-related increase in body weight (at 10 mg/kg: 41.55 ± 2.01, *P* < 0.001) comparable in magnitude to the effect of the IIB-Fc:IIB-Fc homodimer (38.47 ± 1.49, *P* < 0.001) (Fig. 4A). Similarly, IIB-Fc:ALK4-Fc treatment caused a significant increase in gastrocnemius weight (at 10 mg/kg: 234.4 ± 9, *P* < 0.001) and a significant reduction in body fat (at 10 mg/kg: − 3.34 ± 0.45, *P* < 0.001) comparable in magnitude to changes caused by IIB-Fc:IIB-Fc (gastrocnemius weight: 230.6 ± 8.66, *P* < 0.001; body fat: − 3.51 ± 0.33, *P* < 0.001) (Fig. 4B,C), consistent with previous observations^35,37^. These tissue alterations in the presence of IIB-Fc:ALK4-Fc are likely achieved, at least in part, through hypertrophy of individual muscle fibers, and have been reported to accompany enhanced muscle twitch force^35^. Interestingly, the selectively diminished binding to BMP9 and BMP10 by IIB-Fc:ALK4-Fc (Fig. 2B, Table 1) did not appreciably alter its effects on these endpoints. This result could argue against major roles of these BMPs on homeostasis of skeletal muscle or adipose tissue under the conditions tested, although BMP9 has been associated with hepatic function, insulin resistance, obesity, and regulation of energy balance^38–42^. An important distinction between the two fusion proteins emerged when comparing bone mineral density, as treatment with IIB-Fc:ALK4-Fc did not produce a significant increase in this parameter when compared with vehicle (at 10 mg/kg: 11.61 ± 0.94, *P* = 0.32), whereas IIB-Fc:IIB-Fc did (14.29 ± 1.69, *P* = 0.02) (Fig. 4D). Overall, the similar effects of IIB-Fc:ALK4-Fc heterodimer and IIB-Fc:IIB-Fc homodimer on body weight and body composition accord well with the shared high affinities of these agents for the SMAD2/3-pathway ligands activin A, activin B, GDF8, and GDF11 (Fig. 2B, Table 1).

**Figure 5:**
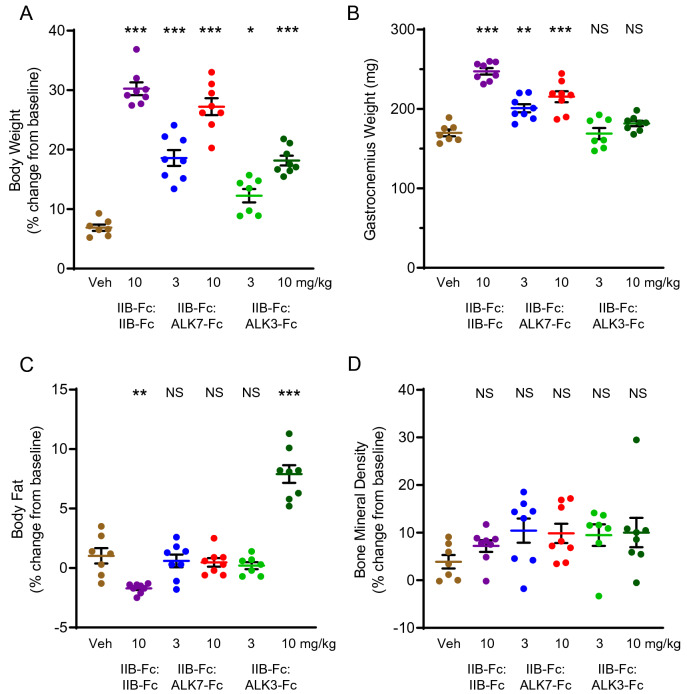
Activity of IIB-Fc:ALK7-Fc and IIB-Fc:ALK3-Fc heterodimers in wild-type mice. Twelve-week-old wild-type C57BL/6 mice were injected s.c. with IIB-Fc:IIB-Fc (*n* = 8, 10 mg/kg), IIB-Fc:ALK7-Fc (*n* = 8, either 3 mg/kg or 10 mg/kg), IIB-Fc:ALK3-Fc (*n* = 7, 3 mg/kg or *n* = 8, 10 mg/kg), or vehicle control (*n* = 7, PBS) twice weekly for 28 days. (**A**) Percentage change in mouse total body weight from day -1 to day 28. (**B**) Weight of the gastrocnemius muscle isolated by dissection on day 28. (**C**) Percentage change in total fat mass normalized to body weight from day -1 to day 27 as assessed by NMR. (**D**) Percentage change in bone mineral density from day -1 to day 27 as assessed by DXA. Data are means ± SEM. See Supplemental Table S3 for associated statistics. *Veh* vehicle; *mg/kg* milligrams per kilogram; *NS* not significant. *P < 0.05, **P < 0.01, ***P < 0.001 vs. vehicle.

Based on SPR analysis, IIB-Fc:ALK7-Fc resembles IIB-Fc:ALK4-Fc in its ligand sequestration profile (Fig. 2C) but notably shows reduced sequestration of activin A, a selective change which could lead to differences in activity between the two agents in vivo. When evaluated in wild-type mice, IIB-Fc:ALK7-Fc produced a dose-dependent increase in body weight (at 10 mg/kg: 27.22 ± 1.42, *P* < 0.001) and gastrocnemius weight (at 10 mg/kg: 215.5 ± 7.03, *P* < 0.001) closely resembling effects of the IIB-Fc:IIB-Fc homodimer (body weight: 30.24 ± 1.07, *P* < 0.001; gastrocnemius weight: 247.4 ± 4.05, *P* < 0.001) (Fig. 5A,B). Notably, IIB-Fc:ALK7-Fc did not alter body fat percentage relative to vehicle (at 10 mg/kg: 0.48 ± 0.36, *P* = 0.89) (Fig. 5C), unlike either the IIB-Fc:IIB-Fc homodimer (− 1.7 ± 0.15, *P* < 0.01) (Fig. 5C) or the IIB-Fc:ALK4-Fc heterodimer (Fig. 4C), both of which reduced fat while increasing muscle weight. This result suggests that one or more ligands with altered affinity for IIB-Fc:ALK7-Fc compared with IIB-Fc:IIB-Fc play a role in promoting maintenance of fat mass under these conditions, which is intriguing given that ALK7 is implicated in adipose tissue homeostasis^43,44^. Bone mineral density was not affected significantly by IIB-Fc:ALK7-Fc (at 10 mg/kg: 9.85 ± 2.03, *P* = 0.23) or IIB-Fc:IIB-Fc treatment (7.2 ± 1.25, *P* = 0.74) under these conditions, perhaps due to high intragroup variability (Fig. 5D). Together, these results indicate that treatment with IIB-Fc:ALK7-Fc evokes only a subset of the effects observed after treatment with the IIB-Fc:IIB-Fc homodimer, consistent with its ligand binding profile, and that a reduction in body fat percentage need not accompany increased body weight and muscle weight under the conditions examined.

The IIB-Fc:ALK3-Fc heterodimer exhibits a ligand-binding profile markedly different from that of the IIB-Fc:IIB-Fc homodimer, including exceptionally strong binding to BMP2 and BMP4, increased binding to BMP6 and BMP7, and reduced binding to BMP9 and BMP10 (Fig. 2D). When evaluated in wild-type mice, IIB-Fc:ALK3-Fc produced a distinctive biologic activity profile as well. Specifically, IIB-Fc:ALK3-Fc caused a dose-dependent increase in total body weight (at 10 mg/kg: 18.17 ± 0.82, *P* < 0.001) that was approximately half the magnitude of the weight gain caused by IIB-Fc:IIB-Fc at the same dose level (30.24 ± 1.07, *P* < 0.001) (Fig. 5A). However, in striking contrast with the homodimer, IIB-Fc:ALK3-Fc increased rather than decreased body fat percentage at the 10 mg/kg dose (7.9 ± 0.74, *P* < 0.001) (Fig. 5C) without altering gastrocnemius weight (181.8 ± 3.36, *P* = 0.38) (Fig. 5B) or bone mineral density (10.03 ± 3.08, *P* = 0.21) (Fig. 5D). Interestingly, the activity profile of IIB-Fc:ALK3-Fc differed also from the ALK3-Fc homodimer, ALK3-Fc:ALK3-Fc, which altered neither body weight nor body fat percentage when tested under equivalent conditions at the same dose level (Supplemental Fig. S3). The heterodimeric construct IIB-Fc:ALK3-Fc thus possesses an activity profile in wild-type mice distinct from either of its corresponding homodimeric traps. These data furthermore suggest that IIB-Fc:ALK3-Fc increases body weight mainly through increased fat mass and not increased muscle mass, and imply that signaling by BMP2 or BMP4 could regulate adipose tissue homeostasis under the conditions tested.

## Discussion

The TGF-β superfamily ligands collectively regulate a wide spectrum of biological processes in animals, from developmental patterning and cellular specification to homeostatic maintenance of adult tissue composition in all major organs. The 33 superfamily members in humans are thought to have diversified from a single ancestral secreted ligand, giving rise to BMPs, GDFs, activins and inhibin, the TGF-βs, and others, with each subfamily acquiring distinctive structural specializations allowing for diversification of functions^45^. However, similarities of molecular structure often permit closely related ligands to engage common receptors and thereby function in a partially redundant manner when one or more ligands are disabled or removed. Although some authors have suggested that ligand redundancy confers robustness to biological systems, recent work indicates that overlapping functionalities allow for complex information to be encoded by combinations of ligands^4–6^. A principal challenge for superfamily-directed therapies is therefore to neutralize groups of ligands involved in pathological signaling while sparing other related ligands with important activities.

Natural selection generated molecules capable of discriminating groups of TGF-β superfamily ligands—the seven type I and five type II transmembrane receptor kinases—that can be repurposed for therapeutic applications. The receptor ECD, when separated from the transmembrane domain and made soluble, can promote a dominant negative effect by sequestering ligands from functional endogenous cell surface receptors. An immunoglobin Fc domain fused to the receptor ECD can further prolong the half-life and functional longevity of the chimeric molecule^24,46^. Many such homodimeric ECD-Fc molecules have been evaluated as drug candidates for diverse pathological conditions, including those comprising either type I^32,47,48^ or type II^49^ receptor homodimers. Multiple homodimeric Fc-fusion ligand traps have now been approved for therapeutic use in humans^50^, including the immune-suppressing agent etanercept (based on tumor necrosis factor receptor 2)^51^ and the erythroid maturation agent luspatercept (a modified ActRIIB-Fc homodimer)^52,53^.

Combinatorial signaling by TGF-β superfamily ligands and receptors is complex^5^, but its transduction pathways are organized by some general principles. Whereas the type II receptors ActRIIA and ActRIIB bind ligands of both canonical SMAD signaling branches, downstream signaling events largely depend on which type I receptor is recruited into an active signaling complex. We reasoned that by pairing one type II ECD with one type I ECD together in a heterodimer, we could narrow the range of targeted ligands from that of a type II homodimer and exert greater control over subsequent signaling events. This prediction was borne out by in vitro biochemical experiments (Fig. 2) and cell-based reporter assays (Fig. 3). Pairing of one ActRIIB arm with either ALK4 or ALK7, receptors with high affinity for SMAD2/3 ligands, resulted in greatly decreased binding of the heterodimer with the SMAD1/5/8 ligands BMP4, BMP9, and BMP10. These results support one potential strategy for effectively targeting muscle regulation through activin and GDF inhibition while avoiding vascular side-effects associated with BMP9 deficiency. Indeed, IIB-Fc:ALK4-Fc was found previously to spare BMP9-dependent activities in a retinal vessel outgrowth assay^35^.

Experimental results obtained for ligand traps in these simplified in vitro systems are seldom fully predictive of effects seen when the trap molecule is placed into an organismal context. For the TGF-β superfamily in particular, context is critically important for determining the cellular or tissue response to any individual ligand—the most well-known example being the dual tumor-suppressive and tumor-promoting functions of TGF-β1^54,55^. The superfamily ligands are subject to extensive regulation by secreted antagonists that can vary in spatiotemporal abundance^56^, activate a range of additional non-canonical signal transduction cascades^57^, act as competitive receptor antagonists^58^, and potentially signal alternately through either SMAD2/3 or SMAD1/5/8 canonical pathways depending on the availability of switching factors—a phenomenon referred to as lateral signaling^59–61^. In addition, it is now recognized that competition for receptor occupancy between high- and low-affinity superfamily ligands can influence cellular responses, such that activin-class molecules of the SMAD2/3 pathway could mask the effects of lower affinity SMAD1/5/8-activators when both are present^6^. Thus, a selective trap neutralizing a subset of ligands in vivo perturbs a complex system of cross-interacting components not modeled by homogenous cell populations in culture, underscoring the importance of screening novel trap molecules in animal models of normal physiology and disease.

The IIB-Fc:IIB-Fc homodimer and IIB-Fc:ALK4-Fc heterodimer promote muscle growth *in vivo*^35^, likely by sequestering multiple factors implicated in the negative regulation of muscle size including activin A, activin B, GDF8, and potentially GDF11^62,63^. Interestingly, these ligand traps also decreased body fat as a percentage of body weight by approximately 4% in the present study (Fig. 4C), raising the question of whether this effect is due to loss of direct signaling to adipose cell types or is instead a secondary consequence to increased skeletal muscle mass. Recent work showed that ablation of ActRIIA and ActRIIB selectively in mouse myofibers causes muscle hypertrophy, decreased body fat, and decreased circulating glucose levels, providing evidence that the reduced fat content in these animals is a non-cell-autonomous effect mediated by enhanced muscularization and associated metabolic shifts^8^. However, inverse changes in muscle mass and fat mass were not observed in the present study when wild-type mice were treated with the IIB-Fc:ALK7-Fc heterodimer, which binds activin B as effectively as the IIB-Fc:IIB-Fc homodimer but has decreased affinity for the other tested ligands (Fig. 2C). Instead, treatment with the heterodimer containing ALK7 increased muscle but did not alter body fat (Fig. 5B,C). These results suggest either that the extent of muscle increase in the treated animals was insufficient to measurably shift fat content or that one or more ligands bound by IIB-Fc:IIB-Fc but spared by IIB-Fc:ALK7-Fc, such as activin A (Figs. 2C, 3A), might regulate adipose tissue size in mice.

The most surprising in vivo results were obtained following treatment with the IIB-Fc:ALK3-Fc heterodimeric trap. This molecule was found to increase body weight without a concomitant increase in muscle, as assessed by gastrocnemius weight. We speculate that increased body weight in these mice is due mainly to the 7.5% increase in body fat content (Fig. 5C). The ALK3-containing heterodimer binds BMP2 and BMP4 ligands with very high affinity—consistent with the important role for ALK3 in mediating SMAD1/5/8 signaling—but has reduced affinity for SMAD2/3 ligands (Fig. 2D). Our results suggest that BMP2 and BMP4 negatively regulate fat content in vivo, that one or more SMAD2/3-activating ligands promote fat accumulation, or both. However, these possibilities would contrast sharply with previous findings^64,65^. Cell culture studies generally support a model in which SMAD1/5/8 signaling promotes proliferation of adipocytes, whereas SMAD2/3 signaling suppresses adipocyte formation^64^. The SMAD1/5/8 small molecule inhibitor LDN-193189, for example, blocks adipogenesis in the 3T3-L1 cell culture model^65^. The interesting contradictions raised by our present in vivo experiments require further investigation, particularly given the emerging role of BMP4 in metabolic disorders^66^. It is possible that the IIB-Fc:ALK3-Fc ligand trap could allow for other more potently adipogenic SMAD1/5/8-related ligands to operate by removing competition by BMP2 and BMP4.

The BMP ligands are named for their well-documented ability to induce bone formation *in vivo*^67^. Strikingly, however, both the targeted disruption of *Bmpr1a* (encoding ALK3) in bone cells^68,69^ and BMP neutralization with a homodimeric ALK3-Fc:ALK3-Fc ligand trap^47^ result in increased bone mass in mice. The mechanisms underlying these surprising effects are not understood, although it has been suggested that BMP2 and BMP4 blockade with ALK3-Fc:ALK3-Fc could relieve inhibition of bone-promoting signaling by Wnt ligands^47^. In addition, homodimeric ligand traps based on the type II receptors ActRIIA^70–73^ and ActRIIB^8,74–77^ have also been found to increase bone mass, raising the possibility that signaling through both SMAD2/3 and SMAD1/5/8 branches could negatively regulate bone formation in vivo. Furthermore, ActRIIB-based traps appear to increase bone formation despite sequestering BMP9, which is otherwise considered to be highly osteogenic^78,79^. Here, we examined one parameter of bone biology, bone mineral density, as part of an initial screen for in vivo pharmacological activities. Systemic treatment with the IIB-Fc:IIB-Fc homodimer facilitated a modest increase to bone mineral density (Fig. 4D), consistent with previous reports, but this effect was not observed across all experimental replicates (Fig. 5D). The ALK3-, ALK4-, and ALK7-containing heterodimers likewise caused no apparent effect upon bone mineral density, at least at the tested standard doses of 3 and 10 mg/kg. It remains possible that small group differences were missed in our study due to lack of adequate statistical power but could be revealed by larger population sizes. Further studies will be required to titrate maximal effects in vivo and to identify any potential signaling effects by these heterodimeric traps upon osteoblasts and osteoclasts, the key bone-remodeling cell types^67^.

The possible advantages offered by dimerization or oligomerization of receptor domains for ligand trap efficacy have been explored in multiple biological contexts. The large family of cytokine ligands, which includes potent immuno-modulatory molecules such as interleukin (IL)-1, IL-4, IL-6, and IL-13, represents an important therapeutic target. By analogy with the TGF-β superfamily of ligands, the cytokines also assemble heteromeric complexes of cell surface receptors whose molecular composition is driven largely by the cytokine^80^. In pioneering work, Economides et al. sought to develop ligand traps comprising heterodimeric pairs of cytokine receptors, which would be expected to sequester their corresponding ligands more effectively than homodimers^81^. However, cellular co-expression of the separate ECD-Fc polypeptide chains produced only limited amounts of the desired heterodimer due to competing formation of the respective homodimers. The authors circumvented this problem by generating inline fusions of two distinct ECDs as a single polypeptide^81^. Other groups have similarly produced inline fusions of vascular endothelial growth factor receptors^82^, tandem repeats of TGF-β receptor ECDs^83^, and heteromers of the TGF-β type II receptor (*TGFBR2*) with either ALK5 (*TGFBR1*)^84^ or betaglycan (*TGFBR3*)^85^. In the present study, we pursued a different strategy by introducing charged amino acids at key positions in the Fc domain to favor preferential pairing of heterodimers through electrostatic complementarity^25–27^. The architecture of these heterodimeric molecules places the ECDs into apposition more akin to their native arrangement upon the plasma membrane surface, which might better promote their cooperative interaction with target ligands.

We utilized this platform of selective heteromeric traps to characterize pairings of ActRIIB with ALK3, ALK4, and ALK7, thereby mimicking native receptor combinations. However, the combinatorial possibilities afforded by this strategy are far greater. It will also be feasible to generate combinations of receptors not known to occur naturally, including heterodimers comprising solely type I receptors or solely type II receptors, which could reveal surprising biochemical and pharmacological activity profiles analogous to those seen with non-native cytokine receptor pairings^80^. Interestingly, there is evidence that TGF-β superfamily ligands occur naturally as heterodimers^86–88^, most notably the dimeric pairing of BMP9 and BMP10 implicated in vascular homeostasis^89^, but the full range and biologic significance of these combinations remains to be determined. In nature and medicine alike, modular recombination can expand a limited tool set to maximize functionality. Innovative, heterodimeric trap molecules might enable researchers to selectively target unique heterodimeric ligands or previously inaccessible combinations of ligands, enabling new opportunities for probing biological mechanisms and new avenues for therapeutic intervention in diverse disease states.

## Methods

### Statement of Ethics

All experimental procedures were performed according to protocols approved by the Acceleron Pharma Institutional Animal Care and Use Committee. All studies were performed in accordance with the relevant guidelines and regulations. This study is reported in accordance with ARRIVE guidelines.

### Construction, expression, and purification of recombinant ligand trap constructs

All TGF-β superfamily receptor domains consist of human amino acid sequences and were made in house except for ALK4-Fc:ALK4-Fc, which was purchased from R&D Systems (Cat #808-AR-100).

The ECDs of ActRIIB (aa 19–134, NP_001097.2), ALK4 (aa 24–126, NP_004293.1), ALK7 (aa 25–113, NP_660302.2), and ALK3 (aa 24–152, NP_004320.2) were amplified by PCR using the cDNA of full length ActRIIB (Invitrogen), ALK4 (Open Biosystems/Thermo Fisher Scientific), ALK7 (GeneCopoeia), and ALK3 (Invitrogen) as templates and then subcloned into vectors containing the human IgG1 Fc domain. Cloning of the IIB-Fc fusion molecule with a murine IgG2a Fc domain was described previously^74^. For the heterodimers IIB-Fc:ALK4-Fc, IIB-Fc:ALK7-Fc, and IIB-Fc:ALK3-Fc, the ECDs of the type II and type I receptors were inserted into two separate mammalian expression plasmids upstream to modified human IgG1 Fc domains that facilitate heterodimerization and minimize homodimerization through electrostatic complementarity. Substitutions of charged amino acids were made at select positions in the Fc domains according to a previously reported strategy^25^. The Fc domain charge modifications were generated by PCR mutagenesis using cDNA of human IgG1 (Invitrogen) as template. For the heterodimeric constructs containing the ECD of type I receptors, enterokinase recognition sequence and 6xHis tag were added C-terminal to the Fc domain by PCR. The ALK7-Fc:ALK7-Fc homodimer contains an enterokinase recognition sequence between the ECD and Fc domains. All ECD-Fc fusion constructs contain the tissue plasminogen activator (tPA) signal sequence at their N-termini.

IIB-Fc:IIB-Fc^23^ and IIB-Fc:ALK4-Fc^35^ were purified from CHO cells as previously described. For generation of heterodimeric constructs, plasmids were transfected at a 1:1 ratio. IIB-Fc:ALK7-Fc and IIB-Fc:ALK3-Fc were expressed by a stable CHO cell pool followed by purification using protein A (Mab SelectSure, Cytiva), nickel-nitrilotriacetic acid (Ni–NTA, Cytiva) with imidazole gradient and an anti-ActRIIB affinity resin, and Q sepharose fast flow (Q FF, Cytiva) ion-exchange chromatography for further concentration and removal of aggregates. The nickel column used for heterodimer purification removes any undesired type II receptor homodimer, whereas the anti-ActRIIB column removes type I receptor homodimers and other impurities. ALK7-Fc:ALK7-Fc was expressed by a stable CHO pool followed by protein A purification and preparative size-exclusion chromatography (SEC). ALK3-Fc:ALK3-Fc was expressed by a stable CHO cell line, followed by purification using protein A and Q sepharose and phenyl sepharose resins. IIB-Fc:IIB-Fc with a murine IgG2a Fc domain was expressed by a stable CHO cell line and purified with protein A and Q ion-exchange chromatography. The final material was dialyzed in PBS, and purity was assessed to be greater than 90% by SDS-PAGE gel with SimplyBlue SafeStain (Thermo Fisher Scientific) and analytical SEC column (Zenix-C SEC-300, Sepax).

### Characterization of ligand binding

All TGF-β superfamily ligands used in this study contained human sequences. Of these, activin A, activin B, GDF8, GDF11 and BMP9 were generated in house, and GDF3, BMP2, BMP4, BMP6, BMP7 and BMP10 were purchased from R&D Systems.

Receptor-ligand binding interactions were determined on a Biacore 8K system (Cytiva Life Sciences) at 37 °C. A series S CM5 sensorchip was immobilized with anti-human Fc antibody (MilliporeSigma) at a density of approximately 5,000 RU on both active and reference flow cells of all channels. Running buffer consisted of HBS-EP + buffer (10 mM HEPES, 150 mM NaCl, 3 mM EDTA and 0.05% v/v Surfactant P20) supplemented with 350 mM NaCl and 0.5 mg/mL bovine serum albumin (BSA). TGF-β superfamily receptor dimers, including four homodimers (IIB-Fc:IIB-Fc, ALK3-Fc:ALK3-Fc, ALK4-Fc:ALK4-Fc and ALK7-Fc:ALK7-Fc) and three heterodimers (IIB-Fc:ALK3-Fc, IIB-Fc:ALK4-Fc and IIB-Fc:ALK7-Fc) were captured at a flow rate of 10 µL/min by the Fc domain on the active flow cells of channels one through seven, respectively, at levels between 100 to 150 RUs. TGF-β superfamily ligands were prepared in two-fold dilution series in Biacore running buffer and then injected at a flow rate of 30 µL/min for 300 s during the association phase of the interaction, and ligand dissociation was followed for 600 s. Varying ranges of ligand concentration were used in setting up the experiment depending on the affinity of each receptor for different ligands. The sensorchip surface was regenerated by injecting 10 mM glycine at pH 1.7 at 100 µL/min for 15 s. Biacore assays were performed in three independent experiments.

All sensorgrams were processed by double referencing—subtraction of the responses from the reference surface and from an average of blank buffer injections—using ligand concentration ranges listed in Supplemental Table S1. To extract kinetic rate constants, a 1:1 Langmuir binding model with a term for mass transport was used for data analysis in most cases. When receptor-ligand interaction displayed biphasic dissociation kinetics and a 1:1 model did not provide an accurate fit of the experimental sensorgrams, a bivalent analyte model was used instead (Table 1). In this model, a bivalent analyte (homodimeric TGF-β superfamily ligand) interacts with captured ‘ligands’ (dimeric TGF-β superfamily receptors) in a two-step binding process that involves initial monovalent 2:1 binding followed by bivalent 2:2 binding. The off-rate derived from the bivalent analyte model best describes the transient binding observed between the ligand and receptor when compared with the 1:1 model. The first binding step is the primary interaction considered in this study^90^.

### Cell-based reporter gene assay

Fc-fusion protein inhibition of reporter activation by activin A, activin B, GDF11 (5 ng/ml), and GDF8 (45 ng/ml) was assessed using A204 cells dually transfected with the pGL3 CAGA12 firefly luciferase reporter plasmid and a control pRL-CMV renilla luciferase reporter plasmid. Inhibition of reporter activation by BMP9 (600 pg/ml) and BMP10 (350 pg/ml) was assessed using T98G cells transfected with the pGL3 BRE firefly luciferase reporter plasmid and a control pRL-CMV renilla luciferase reporter. The CAGA12- and BRE-containing reporter plasmids were generated in-house by incorporating previously reported promoter regions (CAGA12^33^ and BRE^34^) into the pGL3 (Invitrogen) vector.

A204 (ATCC #HTB-82) or T98G (ATCC #CRL-1690) cells were seeded at 1 × 10^5^ or 0.7 × 10^5^ cells per well in a 48 well plate with McCoy’s Medium (ThermoFisher #16600-082) or Eagle's Minimum Essential Medium (EMEM; ATCC #30-2003), respectively, supplemented with 10% fetal bovine serum (FBS), and incubated overnight at 37 °C with 5% CO_2_. The next day, cells were transfected using either X-tremeGENE 9 (Roche #06365809001) for A204 cells or X-tremeGENE HP (Roche #6366546001) for T98G cells by diluting the transfection reagents and reporter plasmids in Opti-MEM (ThermoFisher #31,985–070) and applying the mixture to cells according to manufacturer’s instructions. Ten micrograms of experimental luciferase reporter and 100 ng of control reporter plasmid were used per 48 well plate. Cells were cultured in serum free media containing 0.1% BSA during transfection.

The day after transfection, cells were treated with either ligand alone or ligand combined with Fc-fusion protein in serum free media containing 0.1% BSA for 6 h (for pGL3 CAGA12-luciferase reporter) or overnight (for pGL3 BRE-luciferase reporter). Before being combined with cells, 1:3 serially diluted fusion proteins were incubated with individual ligands for 30 min at 37 °C and 5% CO_2_. Cells were then lysed and chemiluminescence was measured using the Dual-Luciferase Reporter Assay System (Promega E1980) and the Infinite M200 plate reader according to manufacturer’s instructions. The raw activity was presented as relative luciferase unit (RLU) of experimental luciferase activity normalized to the control renilla luciferase activity. In Fig. 3, data are presented as signaling percentage in which the RLU for cells treated with ligand alone was defined as 100%, and the RLU for cells treated with media alone (with 0.1% BSA) was defined as 0%. The IC_50_ was calculated using a four-parameter dose–response curve in GraphPad Prism. Figure 3 depicts percent signaling data from a single representative experiment, whereas Table 2 summarizes IC_50_ data from three independent experimental replicates.

### In vivo experiments and assessment of mouse body composition

All mice in this study were male and were housed in standard cages under 12-h light/12-h dark cycles and fed ad libitum with a standard chow diet. Mice received vehicle control (PBS), IIB-Fc:IIB-Fc homodimer (10 mg/kg), or one of the ALK3-, ALK4-, or ALK7-containing heterodimers (either 3 mg/kg or 10 mg/kg as indicated) via subcutaneous (s.c) injection twice weekly for 28 days. Dose levels for these constructs can be considered approximately equimolar because the constructs possess similar theoretical molecular weights (IIB-Fc:IIB-Fc, 77.69 kDa; IIB-Fc:ALK3-Fc, 80.06 kDa; IIB-Fc:ALK4-Fc, 77.17 kDa; IIB-Fc:ALK7-Fc, 75.34 kDa).

Mice were treated with IIB-Fc:IIB-Fc containing a murine IgG2a Fc domain (Fig. 4) or a human IgG1 Fc domain (Fig. 5) and were compared directly with IIB-Fc:ALK4-Fc–treated mice (Fig. 4) or with IIB-Fc:ALK7-Fc– and IIB-Fc:ALK3-Fc–treated mice (Fig. 5). Substitution of a human IgG1 Fc domain with a murine IgG2a Fc domain did not affect biologic activity of a IIB-Fc:IIB-Fc homodimeric construct (Supplemental Fig. S2). The human IgG1 Fc domain present in homodimeric and heterodimeric constructs is not expected to trigger immunogenicity in experimental mice^91^. Body fat composition was measured by nuclear magnetic resonance (NMR, Bruker MiniSpec) imaging and bone mineral density was measured using dual-energy x-ray absorptiometry (DXA, Lunar PiximusII) on the day prior to first injection (day -1) and the day prior to euthanasia (day 27). Experimental animal ages and group sizes are as indicated in Figs. 4 and 5 legends. Body weight and overall animal health were assessed regularly during the experimental period. Mice were euthanized by CO_2_ asphyxiation on day 28, at which point gastrocnemius muscles were isolated by dissection, flash frozen with liquid nitrogen, and weighed. The gastrocnemius, chosen as a representative muscle, is one of the largest muscles in mice and therefore affected less by dissection variability during tissue harvesting.

### Statistics

All values in Figs. 4 and 5 are expressed as mean ± standard error of the mean (SEM). See Supplemental Tables S2 and S3 for additional descriptive statistics. For in vivo experiments, drug-treatment mean values were compared with vehicle-treated mean values and significance was determined by one-way ANOVA with Dunnett’s post hoc test. *P* values less than or equal to 0.05 were deemed statistically significant. GraphPad Prism version 8 was used for statistical analysis.

## Supplementary Information

Supplementary Information.
