# Supplementary material for: Functionally diverse heteromeric traps for ligands of the transforming growth factor-β superfamily
Source: Sci Rep. 2021 Sep 15;11:18341. doi: 10.1038/s41598-021-97203-9 (PMC8443706; doi:10.1038/s41598-021-97203-9)
Supplement: Supplementary file 1 — Supplementary Information. [file 41598_2021_97203_MOESM1_ESM.pdf]

Functionally diverse heteromeric traps for ligands of the transforming growth factor-β superfamily

Ravindra Kumar[1], Asya V. Grinberg[1,2], Huiming Li[1], Tzu-Hsing Kuo[1], Dianne Sako[1], Lavanya Krishnan[1], Katia Liharska[1,2], Jia Li[1], Rosa Grenha[1], Michelle C. Maguire[1], Steven D. Briscoe[1], R. Scott Pearsall[1,3], Brantley R. Herrin[1], Rajasekhar N.V.S. Suragani[1*], Roselyne Castonguay[1*]

[1]Acceleron Pharma, Cambridge, Massachusetts, USA

[2]Current affiliation: Dragonfly Therapeutics, Waltham, Massachusetts, USA

[3]Current affiliation: Cellarity, Cambridge, Massachusetts, USA

[*]Corresponding authors

rsuragani@acceleronpharma.com

rcastonguay@acceleronpharma.com

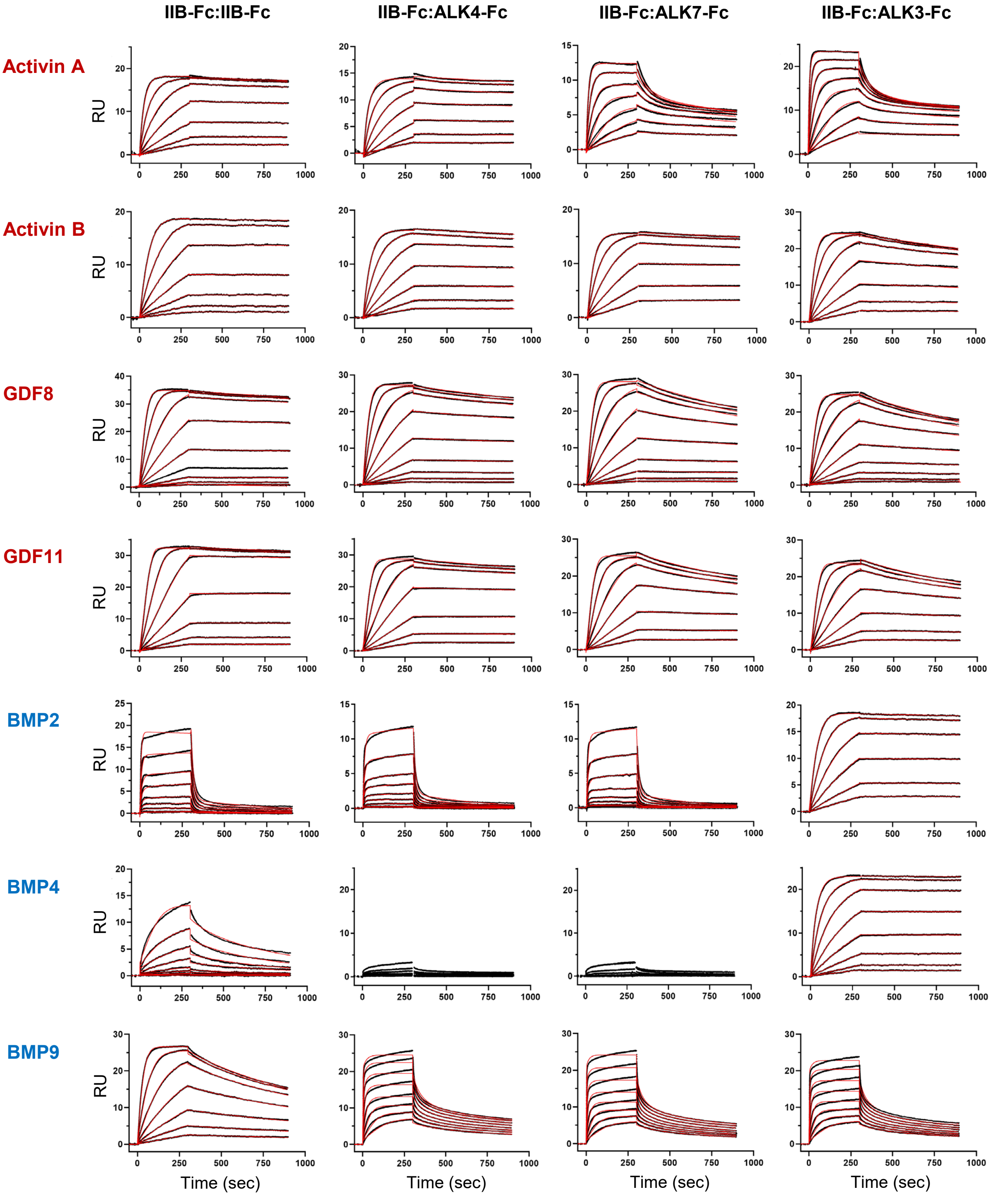

Supplemental Figure 1. Representative SPR sensorgrams for the homodimer IIB-Fc:IIB-Fc and heterodimers IIB-Fc:ALK4-Fc, IIB-Fc:ALK7-Fc, and IIB-Fc:ALK3-Fc evaluated with individual SMAD2/3-pathway ligands (red) and SMAD1/5/8-pathway ligands (blue) to determine values for kinetic parameters shown in Table 1. Sensorgrams (black lines) are overlaid in most cases with fits to a 1:1 interaction model with mass transport limitations or in certain cases (identified in Table 1) with fits to a bivalent analyte model (red lines in all cases). The range of ligand concentrations used for curve fitting are indicated for each construct-ligand combination in Supplemental Table 1. RU, resonance unit.

Supplemental Table 1. Ligand concentration ranges used for curve fitting for all combinations of ligands and constructs.

Ligand	Reference	Heterodimers			Homodimers		
	IIB-Fc: IIB-Fc	IIB-Fc: ALK4-Fc	IIB-Fc: ALK7-Fc	IIB-Fc: ALK3-Fc	ALK4-Fc: ALK4-Fc	ALK7-Fc: ALK7-Fc	ALK3-Fc: ALK3-Fc
Activin A	0.078–5	0.078–5	0.156–20	0.156–20	0.78–100	0.78–100	0.78–100
Activin B	0.078–5	0.078–5	0.156–5	0.156–10	0.39–50	0.39–50	0.39–50
GDF3	0.625–20	0.625–80	0.625–80	0.625–80	0.625–80	0.625–80	0.625–80
GDF8	0.078–20	0.078–20	0.078–20	0.078–20	0.39–100	0.39–100	0.39–100
GDF11	0.078–5	0.078–5	0.078–5	0.078–5	0.39–100	0.39–100	0.39–100
BMP2	0.156–40	0.156–40	0.156–40	0.078–2.5	0.156–40	0.156–40	0.156–20
BMP4	0.156–40	0.156–40	0.156–40	0.078–10	0.156–40	0.156–40	0.156–40
BMP6	0.078–10	0.312–40	0.312–40	0.078–10	0.312–40	0.312–40	0.312–40
BMP7	0.312–20	0.312–20	0.312–20	0.078–10	0.312–20	0.312–20	0.312–20
BMP9	0.078–5	0.78–100	0.78–100	0.78–100	0.78–100	0.78–100	0.78–100
BMP10	0.039–2.5	0.039–5	0.039–5	0.039–2.5	0.39–100	0.39–100	0.39–100

Units are expressed as nM.

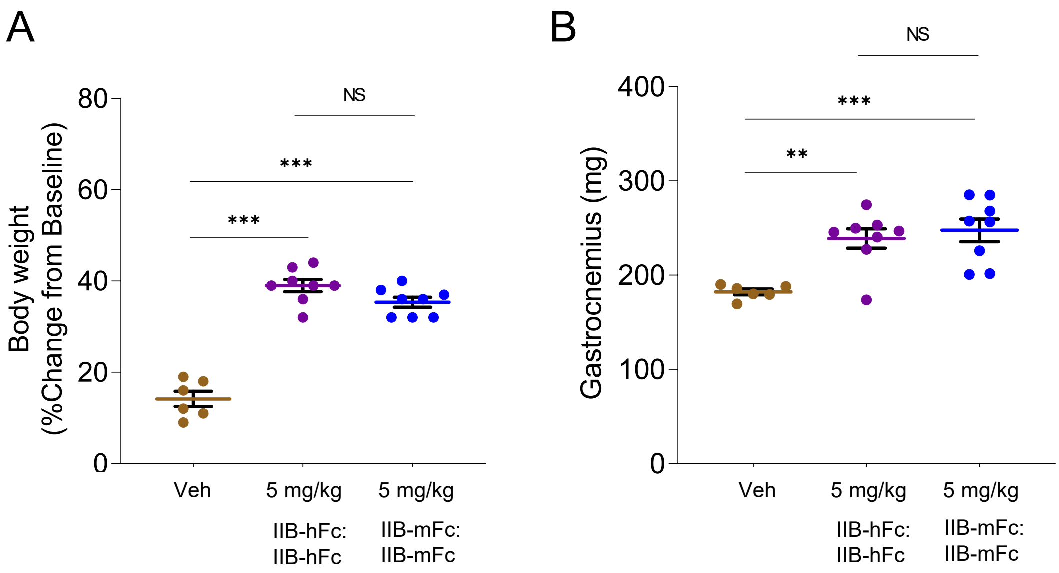

Supplemental Figure 2. Activity of IIB-hFc:IIB-hFc (human Fc) and IIB-mFc:IIB-mFc (mouse Fc) homodimers in wild-type mice. Twelve-week-old wild-type C57BL/6 mice were injected s.c. with IIB-hFc:IIB-hFc (n = 8, 5 mg/kg), IIB-mFc:IIB-mFc (n = 8, 5 mg/kg), or vehicle control (n = 6, PBS) twice weekly for 8 weeks. (**A**) Percentage change in mouse total body weight from baseline to end of 8 weeks. (**B**) Weight of the gastrocnemius muscle isolated by dissection on study termination at the end of 8 weeks. Veh, vehicle; mg/kg, milligrams per kilogram; NS, not significant. **$P < 0.01$, *** $P < 0.001$.

Supplemental Table 2. Supplemental statistics for Figure 4.

	Parameter	Vehicle	IIB-Fc: IIB-Fc: 10 mg/kg	IIB-Fc: ALK4-Fc 3 mg/kg	IIB-Fc: ALK4-Fc 10 mg/kg
	Number of animals	9	9	9	9
Body weight (% change)	Mean	14.95	38.47	33.42	41.55
	Std. dev.	3.91	4.48	8.84	6.03
	SEM	1.3	1.49	2.95	2.01
	Lower 95% CI	11.95	35.03	26.62	36.91
	Upper 95% CI	17.95	41.92	40.21	46.18
Gastrocnemius weight	Mean	152.9	230.6	209.3	234.4
	Std. dev.	13.1	25.97	19.74	27
	SEM	4.37	8.66	6.58	9
	Lower 95% CI	142.9	210.7	194.2	213.6
	Upper 95% CI	163	250.6	224.5	255.2
Body fat (% change)	Mean	0.22	-3.51	-3.74	-3.34
	Std. dev.	1.91	1	1.18	1.36
	SEM	0.64	0.33	0.39	0.45
	Lower 95% CI	-1.25	-4.28	-4.65	-4.39
	Upper 95% CI	1.69	-2.74	-2.84	-2.3
Bone mineral density (% change)	Mean	8.58	14.29	11.18	11.61
	Std. dev.	2.81	5.06	5.62	2.83
	SEM	0.94	1.69	1.88	0.94
	Lower 95% CI	6.42	10.4	6.85	9.43
	Upper 95% CI	10.74	18.17	15.5	13.79

Std. dev., standard deviation; SEM, standard error of the mean; CI, confidence interval

Supplemental Table 3. Supplemental statistics for Figure 5.

	Parameter	Vehicle	IIB-Fc: IIB-Fc: 10 mg/kg	IIB-Fc: ALK7-Fc 3 mg/kg	IIB-Fc: ALK7-Fc 10 mg/kg	IIB-Fc: ALK3-Fc 3 mg/kg	IIB-Fc: ALK3-Fc 10 mg/kg
	Number of animals	7	8	8	8	7	8
Body weight (% change)	Mean	6.89	30.24	18.58	27.22	12.26	18.17
	Std. dev.	1.4	3.04	3.77	4.01	2.99	2.31
	SEM	0.53	1.07	1.33	1.42	1.13	0.82
	Lower 95% CI	5.6	27.7	15.42	23.87	9.5	16.24
	Upper 95% CI	8.18	32.78	21.73	30.57	15.03	20.1
Gastrocnemius weight	Mean	169.8	247.4	200.9	215.5	169	181.8
	Std. dev.	11.35	11.46	14.63	19.88	18.6	9.49
	SEM	4.29	4.05	5.17	7.03	7.03	3.36
	Lower 95% CI	159.3	237.8	188.7	198.8	151.8	173.8
	Upper 95% CI	180.3	256.9	213.1	232.1	186.2	189.7
Body fat (% change)	Mean	1.03	-1.7	0.6	0.48	0.2	7.9
	Std. dev.	1.72	0.41	1.48	1.02	0.78	2.1
	SEM	0.65	0.15	0.52	0.36	0.29	0.74
	Lower 95% CI	-0.56	-2.04	-0.64	-0.38	-0.52	6.14
	Upper 95% CI	2.62	-1.36	1.84	1.33	0.92	9.66
Bone mineral density (% change)	Mean	3.89	7.2	10.44	9.85	9.48	10.03
	Std. dev.	3.77	3.52	7.15	5.74	6	8.7
	SEM	1.43	1.25	2.53	2.03	2.27	3.08
	Lower 95% CI	0.4	4.25	4.46	5.06	3.93	2.76
	Upper 95% CI	7.38	10.14	16.41	14.65	15.03	17.31

Std. dev., standard deviation; SEM, standard error of the mean; CI, confidence interval

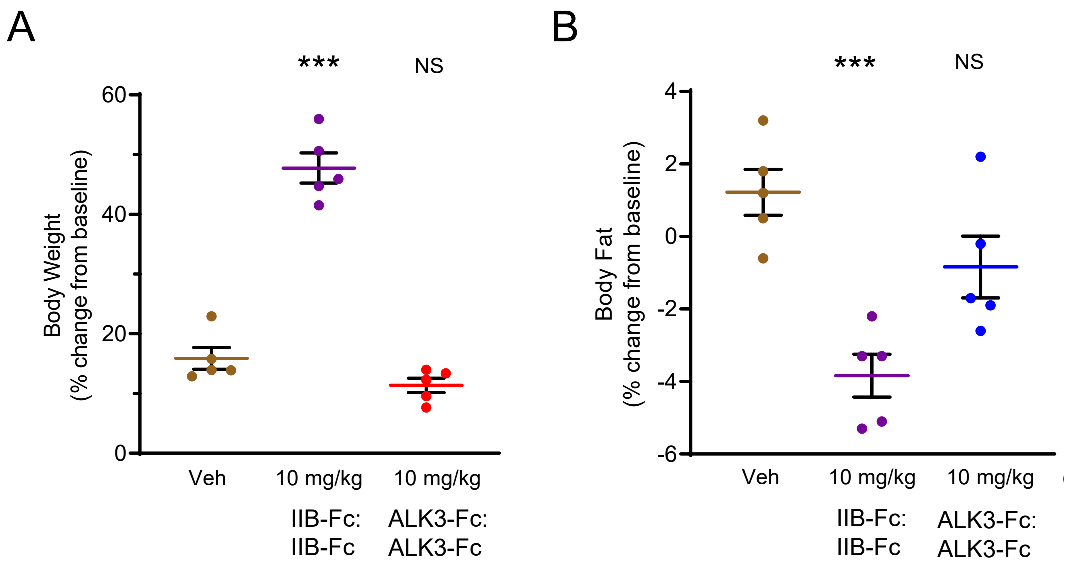

Supplemental Figure 3. Activity of ALK3-Fc:ALK3-Fc homodimer in wild-type mice. Ten-week-old wild-type C57BL/6 mice were injected s.c. with IIB-Fc:IIB-Fc (n = 5, 10 mg/kg), ALK3-Fc:ALK3-Fc (n = 5, 10 mg/kg), or vehicle control (n = 5, PBS) twice weekly for 28 days. (**A**) Percentage change in mouse total body weight from day -1 to day 28. (**B**) Percentage change in total fat mass normalized to body weight from day -1 to day 27 as assessed by NMR. Data are means ± SEM. Veh, vehicle; mg/kg, milligrams per kilogram; NS, not significant. *** $P < 0.001$.
